# Wabash Valley Dental Association

**Published:** 1864-11

**Authors:** 


### Proceedings of Societies.

---

### WABASH VALLEY DENTAL ASSOCIATION.

The Second Semi-Annual session convened in Crawfordsville, Ind., in the rooms of Dr. Totten, October 25, 1864.

Dr. A. M. Moore, President, in the chair.

Minutes of previous meeting read and approved.

The following gentlemen were duly elected members of the Society: Drs. Knapp, Gailey, Manlove, Totten and Clark, who were presented to the Society by Drs. Cunningham and Hurd. On motion of Dr. Budd, Messrs. Field and Lund were elected honorary members.

Next in order was the reading of the President's Address.

On motion of Dr. Snoddy, the address was received, and a copy requested for publication in the Dental journals.

The next business in order being the discussion of Alveolar Abscess. Drs. Knapp, Hurd and Moore were the principal participants.

Adjourned, to meet at 7 P. M.

### EVENING SESSION.

The discussion of Alveolar Abscess was continued, interesting and spirited.

Adjourned to meet at 9 A. M. to-morrow.

### WEDNESDAY MORNING SESSION.

President A. M. Moore in the chair.

Compound Fillings being next in order, the subject elicited a good deal of interest, and proved a very interesting topic for discussion.

Separating Teeth and Professional Etiquette were discussed at considerable length.

On motion of Dr. Hurd, three persons were appointed to read desertations at next meeting.

The Chair appointed Drs. Hurd, Winslow and Knapp.

Received the following report from the Treasurer:

| | |
|---|---|
| Amount Received for Initiation Fee, - - - | $16 00 |
| Amount Paid out, - - - - - - - | 5 00 |
| Remaining in Treasury, - - | $11 00 |

On motion of Dr. Burt, an order be drawn on the Treasurer for an amount sufficient to procure appropriate books for the Secretary of the Society, was adopted.

Meeting adjourned to meet in the city of Fort Wayne, on the 2d day of May, 1865.

Taking into consideration that our Society has scarcely completed its organization, I feel a delicacy in reporting the proceedings in detail; but claiming to have good material to carry out the work we have undertaken. I feel confident in saying that the transactions of this Society shall be an honor to the dental profession in the West.

W. H. PIFER, *Sec'y.*

ADDRESS BY A. M. MOORE, PRESIDENT.

We have great reason to congratulate ourselves when we consider the happy circumstances under which we assemble in our associate capacity. We are in the midst of tremendous events which daily transpire, and which for near four years have threatened the very existence of our government, and all our national, civil, and political institutions. As a nation we have been severely punished, by national calamity for national sin, and few houses, North or South, have not been called to mourn the loss of a father, brother, son, or other near relative, as a sacrifice on the altar of our national calamity, But when I look around me to-day, it is with the livliest gratitude that I behold not one absent whose body has been left to moulder and decay on the bloody field of strife. That this is so is not because the members of our profession have stood aloof from the conflict which calls every friend of his country, and of civil liberty everywhere, to assist in this mightiest of all struggles for the disenthrallment of man from the tyranny of his fellow man. By no means. Many of our profession have entered the field as surgeons, and hold a high rank among those who by their professional labors, have added to the strength and value of our achievmeets. Not a few have entered the army in command of regiments, companies, and parts of companies, and many more have left the busy labors of the operating room and laboratory to swell the mighty mass of freemen who have crowded the ranks as privates to fight the battles of our country.

To-day, gentlemen, we have met together under such happy auspices in our professional capacity, to consult together for our mutual good. And while doing so we must look well to our influence and doings. The little mountain rill which threads its way like a silver thread among the hills and through the valleys, has in itself considered but meek pretentions to influences. But let it pass along. Soon it mingles with other rills, and they with others, until finally with united force, they urge their way as the mighty river to the bosom of the vast ocean, which bears on its bosom the commerce of the world. So it is with our association. It is not confined in influence to this beautiful valley, but mingling in effort with other associations, we throw our weight into the laps of State Associations, and they in turn into National associations, until finally the action of our association, however humble we in our native modesty may esteem them, tell with power on the interests of the world in a professional point of view. How important therefore, gentlemen, that dignity, courtesy and devotion to the supreme good of our noble calling should control our actions. Ours is a noble calling, not a whit behind the noblest of all professions in point of importance to the human family. The time was when barbers and blacksmiths were considered amply sufficient to perform all operations in dental surgery. But now it is as necessary to have an educated dentist as an educated physician. And indeed I may say with truth that the people are less willing to trust themselves to an ignorant pretender in dentistry than they are to trust their lives and health in the hands of charlatins. The civilization of mankind in its highest sense, requires all the appliances of nature and art to add to and preserve the beauty and integrity of the human form. And certainly nothing adds more to the end than the science of dentistry. Hence the very wants of mankind have compelled our seniors to establish a separate branch of the healing art devoted exclusively to the dental organs. The building up and endowments of schools of art and literature expressly and solely for its interests has been a mighty work, and in consequence no profession has made such rapid strides in the same period of time. And surely none stand higher and fairer in the public mind as a necessary means of doing the greatest amount of good to our race in polite and elevated society. It becomes us, therefore, to feel the full importance of our calling. And by a diligent and constant devotion to the study of its various parts to seek to make each one of us ourselves eminent. We have no resting place in any of the branches that we may quietly set down with the satisfaction that all has been accomplished. None reach so high in eminence that if he rests satisfied with his attainments some one more diligent and persevering than he will quietly bear away his "palm of greatness."

Our motto is "upward and onward." Excelsior is written on our temple, and no one is worthy to enter its sacred portals who does not seek the highest interests of his calling.

